# Dairy intake in relation to cardiovascular disease mortality and all-cause mortality: the Hoorn Study

**DOI:** 10.1007/s00394-012-0363-z

**Published:** 2012-05-05

**Authors:** Marieke A. van Aerde, Sabita S. Soedamah-Muthu, Johanna M. Geleijnse, Marieke B. Snijder, Giel Nijpels, Coen D. A. Stehouwer, Jacqueline M. Dekker

**Affiliations:** 1 Division of Human Nutrition, Wageningen University, PO Box 8129, 6700 EV Wageningen, The Netherlands; 2 Department of Public Health, Academic Medical Center, University of Amsterdam, Amsterdam, The Netherlands; 3 EMGO Institute for Health and Care Research, VU University Medical Center, Amsterdam, The Netherlands; 4 Department of Internal Medicine, Academic Hospital Maastricht, Maastricht, The Netherlands; 5 Cardiovascular Research Institute Maastricht, Maastricht University Medical Center, Maastricht, The Netherlands

**Keywords:** Dairy, Cardiovascular diseases, Mortality, Prospective

## Abstract

**Purpose:**

Existing data from prospective cohort studies on dairy consumption and cardiovascular diseases are inconsistent. Even though the association between total dairy and cardiovascular diseases has been studied before, little is known about the effect of different types of dairy products on cardiovascular diseases (CVD). The objective of this study was to examine the relationship between (type of) dairy intake and CVD mortality and all-cause mortality in a Dutch population.

**Methods:**

We examined the relationship between dairy intake and CVD mortality and all-cause mortality in 1956 participants of the Hoorn Study (aged 50–75 years), free of CVD at baseline. Hazard ratios with 95 % CIs were obtained for CVD mortality and all-cause mortality per standard deviation (SD) of the mean increase in dairy intake, with adjustment for age, sex, BMI, smoking, education, total energy intake, alcohol consumption, physical activity, and dietary intakes.

**Results:**

During 12.4 years of follow-up, 403 participants died, of whom 116 had a fatal CVD event. Overall dairy intake was not associated with CVD mortality or all-cause mortality. Each SD increase in high-fat dairy intake was associated with a 32 % higher risk of CVD mortality (95 % CI; 7–61 %).

**Conclusion:**

In this prospective cohort study, the intake of high-fat dairy products was associated with an increased risk of CVD mortality.

## Introduction

Cardiovascular diseases (CVD) are a major cause of death worldwide, claiming 17 million lives a year. Risk factors for CVD are smoking, overweight, hypertension, high plasma cholesterol levels, and diabetes mellitus [1]. Dietary recommendations for the prevention of CVD include reduced intake of saturated fat intake, for example by means of consuming low-fat dairy instead of whole-fat dairy [1].

Low-fat dairy foods are also one of the main components of the Dietary Approaches to Stop Hypertension (DASH) diet, which has been shown to lower blood pressure [2]. However, the beneficial effect could not be ascribed to the low-fat dairy products only, since the diet comprised many other components that are beneficial to health, such as fibers, fruits, and vegetables. Fermented dairy products may also have a beneficial effect on CVD, by reducing the cholesterol concentrations in the blood [3].

Among results of studies assessing the relationship between overall dairy intake and CVD, discrepancy exists. Some observational studies reported no association between dairy intake and CVD [4–8], while another study found an increased risk on developing CVD [9]. There are furthermore studies that found an inverse association between dairy intake and CVD [10–12]. There is growing evidence for a beneficial effect of dairy consumption on blood pressure [9, 13–15].

Despite the abundance of information, it is difficult to draw conclusions from the different studies. Dairy comprises many products, such as milk, cheese, yoghurt, and desserts. It is currently unclear how these specific dairy products like high-fat dairy, low-fat dairy, cheese, or fermented dairy are associated with CVD. This is also supported by review articles and meta-analysis papers [16–21]. Some state that there is no clear evidence that higher dairy food consumption is consistently associated with a higher risk of CVD [17–19]. Others conclude that there is fairly convincing evidence that milk and dairy consumption is associated with an increase in survival in Western communities [20].

Gibson et al. [18] conclude that the studies available for examining the relationship between dairy food consumption and coronary heart disease (CHD) vary too much in design, quality, and dietary assessment methodology. Meta-analyses were mainly dominated by American studies, and often, no distinction was made between the different types of dairy.

Therefore, the purpose of the present study was to determine the relationship between (type of) dairy intake and CVD mortality and all-cause mortality in a Dutch cohort of men and women aged 50–75 years. Total dairy and the different subcategories (high-fat dairy, low-fat dairy, milk, milk and milk products, fermented dairy and cheese) were taken into account. Both CVD mortality and all-cause mortality were used as main outcomes. In the Netherlands, the consumption of dairy is naturally high and a large variety of dairy products is consumed. Only few studies have evaluated the relationship between dairy and dairy categories and the risk of CVD mortality.

## Subjects and methods

### Study population

The Hoorn Study is a Dutch cohort study of diabetes and diabetes complications among the general population, which began in 1989. The study is conducted in 2,484 Caucasian men and women aged 50–75 years. The cohort and baseline measurements are described in detail elsewhere [22].

For this study, 477 subjects were excluded because they had CVD at baseline and 51 subjects were excluded because of missing information on dietary intake. Therefore, the analyses were performed in 1956 subjects (857 men and 1,099 women).

Written consent was obtained from all participants. The study was approved by the Ethics Committee of the VU Medical Center.

### Baseline measurements

Weight and height were measured, and body mass index (BMI) was calculated as weight (kg) divided by height squared (m^2^). Blood pressure was measured on the right arm with a random-zero sphygmomanometer (Hawksley–Gelman Ltd, Lancing, United Kingdom) while subjects were sitting.

Information on lifestyle factors was obtained by a self-administered questionnaire, which was checked by a personal interview. Smoking status was categorized as current smoker, ex-smoker, or non-smoker. Physical activity was expressed in number of hours of physical activity per day. The activities included sports, bicycling, gardening, walking, doing odd jobs, and housekeeping. Alcohol intake was divided into four categories: non-drinker, ≤10, 10–30, and ≥30 g/day. There were three categories of educational level: low, medium, and high.

### Assessment of dairy consumption

To assess dietary intakes of the participants, a 92-item validated semi-quantitative food-frequency questionnaire (FFQ) was used [23], which included the dairy consumption. The participants filled in the questionnaire at home, and the FFQs were checked in the research center for completeness.

Low-fat dairy was defined as milk products with a fat content <2.0/100 g or cheese products with a fat content <20/100 g. High-fat dairy was defined as milk products with a fat content >2.0/100 g or cheese products with a fat content >20/100 g. High-fat dairy was further divided into desserts (yoghurt, curds, custards, and ice-cream) and non-desserts (milk, cheese, porridge, and cream) [24]. The category “milk and milk products” included all kind of milk, yoghurt, coffee creamer, curd, pudding, porridge, and cream. The category “milk” included skimmed, semi-skimmed, and whole milk. The category “fermented dairy” included all fermented products, such as yoghurt, buttermilk, curds, and cheese products. The category “cheese” included all kinds of cheese, that is, soft cheese and hard cheese.

### Outcome measurements

Information on mortality was obtained from the general practitioners and the local hospital. Causes of death were coded according to the International Classification of Diseases, Injuries, and Causes of Death, ninth revision (ICD-9). Fatal CVD was defined as ICD codes 390–459 (diseases of the circulatory system).

Data on fatal CVD were complete until 2005. The mean follow-up in years was 12.4 (24,312 person years).

### Statistical analysis

Baseline characteristics are displayed for the total study population. Cox proportional hazard models were used to estimate hazard ratios (HRs with 95 % CIs) for fatal CVD and all-cause mortality. Because of the limited cases for fatal CHD (*n* = 50) and stroke (*n* = 21), these endpoints were not analyzed separately.

The HRs were estimated per standard deviation (SD) of the mean of dairy product intake.

The basic model was not adjusted for any confounder (model 1). Model 2 included age (continuous) and sex. Subsequently, a multivariate analysis (model 3) was performed with adjustment for age, sex, BMI (continuous), smoking (3 categories), educational level (3 categories), total energy intake (continuous), and alcohol consumption (4 categories). Further adjustments (model 4) were then made for physical activity (continuous) and for intakes of meat, fish, bread, vegetables, fruit, coffee, and tea (continuous).

The statistical analyses were performed by using SAS software (version 9.2; SAS Institute, Cary, NC, USA).

## Results

### Baseline characteristics

Baseline characteristics of the study population are shown in Table 1. The mean age of this population was 61.6 years and 43.8 % was male.

**Table 1:** Mean (±SD) baseline characteristics and dietary intakes of 1956 men and women from the Hoorn Study

Characteristic	Value
*N*	1,956
Age (year)	61.1 (7.2)
Sex (% male)	43.8
BMI (kg/m^2^)	26.5 (3.5)
Physical activity (hour/day)	4.4 (2.7)
Educational level	
Low (%)	80.2
Medium (%)	13.6
High (%)	6.2
Current smoker (%)	31.5
Alcohol (g/day)	9.0 (12.2)
Systolic BP (mmHg)	135 (20)
Diastolic BP (mmHg)	82 (10)
Serum total cholesterol (mmol/L)	6.6 (1.2)
LDL-cholesterol (mmol/L)	4.6 (1.1)
HDL-cholesterol (mmol/L)	1.3 (0.4)
NDD (%)	6.1
Known diabetes (%)	2.8
Hypertension (%)	26.0
Dietary intakes	
Energy intake (kcal/day)	2,072 (587)
Total dairy (g/day)^a^	466.0 (270.9)
High-fat dairy (g/day)^b^	169.3 (178.5)
Low-fat dairy (g/day)^c^	295.3 (260.4)
Fermented dairy (g/day)^d^	218.2 (182.1)
Milk and milk products (g/day)^e^	430.0 (267.5)
Milk (g/dday)^f^	166.3 (202.4)
Cheese (g/day)^g^	30.1 (21.8)
Meat (g/day)	120.8 (59.8)
Fish (g/day)	18.1 (29.0)
Bread (g/day)	128.9 (59.9)
Vegetables (g/day)	108.3 (47.4)
Fruit (g/day)	229.5 (152.3)
Fiber (g/day)	27.2 (7.8)
Coffee (mL/day)	531.5 (290.0)
Tea (mL/day)	296.9 (251.4)
Total fat (g/day)	95.4 (33.2)
Saturated fat (g/day)	40.1 (14.7)
Total protein (g/day)	75.1 (21.2)
Total calcium (mg/day)	1,091 (409)

*BP* blood pressure, *NDD* newly detected diabetes

^a^Includes all dairy products, except butter

^b^Defined as all milk products with a fat content >2.0/100 g or cheese products with a fat content >20 g/100 g

^c^Defined as all milk products with a fat content <2.0/100 g or cheese products with a fat content <20/100 g

^d^Includes all fermented products, such as yogurt, buttermilk, curds, and cheese products

^e^Includes all kinds of milk, yoghurt, coffee creamer, curd, pudding, porridge, and cream (both low-fat and high-fat)

^f^Includes all milk: skimmed, semi-skimmed, and whole milk

^g^Includes soft cheese and hard cheese (both low-fat and high-fat)

### Dairy consumption

**Fig. 1:**
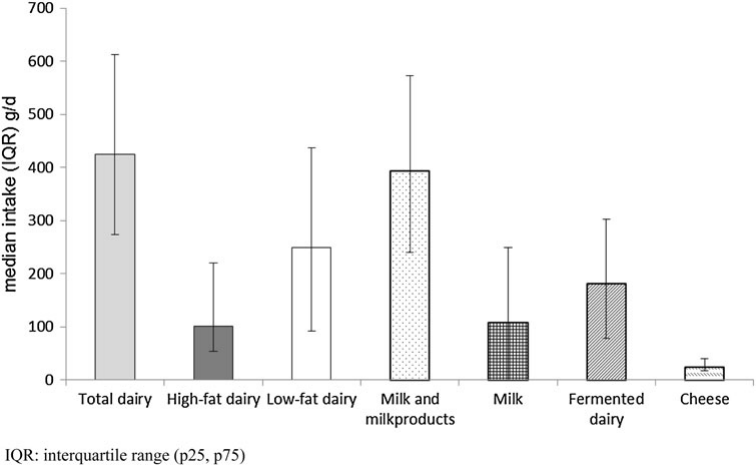
Median intakes and interquartile ranges of dairy products in g/day. *IQR* interquartile range (p 25, p 75)

The median intake of total dairy products in this population was 425 g/day. Median intakes in the total population of high-fat dairy, low-fat dairy, fermented dairy, milk and milk products, milk, and cheese were 101, 250, 181, 394, 108, and 24 g/day, respectively (Fig. 1 for median intakes of dairy products and interquartile ranges).

The SDs of the mean intake (shown in Table 1) were 271 g/day for total dairy, 179 g/day for high-fat dairy, 260 g/day for low-fat dairy, 268 g/day for milk and milk products, 202 g/d for milk, 182 g/day for fermented dairy, and 22 g/day for cheese.

### Dairy intake and fatal CVD

Until January 2005, 403 subjects died, of whom 116 had a fatal CVD event.

No association was found between total dairy intake and CVD mortality (multivariate HR, 1.18; 95 % CI, 0.96, 1.44). For high-fat dairy, however, each SD increase in intake was significantly associated with a 32 % higher risk of CVD mortality (95 % CI, 7, 61 %) (Table 2). Additional adjustment for low-fat dairy increased the HR even further (HR, 1.38; 95 % CI, 1.10, 1.72, data not shown in tables). Further adjustments for LDL-cholesterol, HDL-cholesterol, or hypertension only increased the HR further (data not shown in tables).

**Table 2:** Hazard ratios (HRs) and 95 % CIs of fatal CVD per SD increase of dairy intake

Fatal CVD (*n* = 116)*				
	Model 1^a^	Model 2^b^	Model 3^c^	Model 4^d^
Total dairy^e^	1.07 (0.90, 1.28) *P* = 0.46	1.04 (0.88, 1.23) *P* = 0.65	1.06 (0.88, 1.29) *P* = 0.54	1.18 (0.96, 1.44) *P* = 0.11
High-fat dairy^f^	**1.19 (1.03, 1.39)** *P* = **0.02**	1.16 (1.00, 1.35) *P* = 0.06	**1.19 (1.01, 1.41)** *P* = **0.04**	**1.32 (1.07, 1.61)** *P* = **0.009**
Low-fat dairy^g^	0.93 (0.77, 1.12) *P* = 0.42	0.93 (0.78, 1.12) *P* = 0.45	0.94 (0.78, 1.14) *P* = 0.52	1.01 (0.83, 1.23) *P* = 0.96
Milk and milk products^h^	1.08 (0.91, 1.29) *P* = 0.39	1.04 (0.881, 1.24) *P* = 0.63	1.06 (0.88, 1.29) *P* = 0.54	1.17 (0.96, 1.43) *P* = 0.13
Milk^i^	1.16 (0.99, 1.36) *P* = 0.06	1.10 (0.94, 1.29) *P* = 0.23	1.14 (0.96, 1.34) *P* = 0.13	1.17 (0.98, 1.39) *P* = 0.09
Fermented dairy^j^	0.85 (0.69, 1.04) *P* = 0.11	0.91 (0.75, 1.11) *P* = 0.35	0.90 (0.73, 1.11) *P* = 0.32	1.01 (0.80, 1.27) *P* = 0.97
Cheese^k^	0.89 (0.73, 1.08) *P* = 0.24	0.97 (0.791, 1.19) *P* = 0.77	0.98 (0.79, 1.20) *P* = 0.81	1.09 (0.87, 1.35) *P* = 0.46

The SDs were 271 g/day for total dairy, 179 g/day for high-fat dairy, 260 g/day for low-fat dairy, 268 g/day for milk and milk products, 202 g/day for milk, 182 g/day for fermented dairy, and 22 g/day for cheese

Bold values are statistically significant (*P* < 0.05)

* Due to missings on confounding variables, the number of cases in model 3 is 108 (total: 1,848), and in model 4, this is 93 (total: 1,637)

^a^Unadjusted

^b^Adjusted for age and sex

^c^Adjusted for age, sex, BMI, smoking, educational level, total energy intake, alcohol consumption

^d^Adjusted as for model 1 with additional adjustment for physical activity and intake of meat, fish, bread, vegetables, fruit, coffee, and tea

^e^Includes all dairy products except butter

^f^Defined as all milk products with a fat content >2.0/100 g or cheese products with a fat content >20/100 g

^g^Defined as all milk products with a fat content <2.0/100 g or cheese products with a fat content <20/100 g

^h^Includes all kinds of milk, yoghurt, coffee creamer, curd, cream, porridge, and pudding (both low-fat and high-fat)

^i^Includes all milk: skimmed, semi-skimmed, and whole milk

^j^Includes all fermented products, such as yogurt, buttermilk, curds, and cheese products

^k^Includes soft cheese and hard cheese (both low-fat and high-fat)

When physical activity was omitted from model 4, since 255 participants had missing values on physical activity, the HR for each SD increase in high-fat dairy was 1.24 (95 % CI, 1.03, 1.49, data not shown in tables).

Additional adjustment for saturated fat intake slightly weakened the results (HR, 1.29; 95 % CI, 1.04, 1.59, data not shown in tables), but this association remained statistically significant. Additional adjustments for total fat, protein, or calcium did not change the results (data not shown in tables).

Further division of high-fat dairy products into desserts (33 %) and non-desserts (66 %) demonstrated no statistically significant association between desserts and CVD mortality (multivariate HR per SD (77.8 g/day) increase, 1.10; 95 % CI, 0.90, 1.35). On the other hand, consumption of non-desserts high-fat dairy was significantly associated with an increased risk of CVD mortality (HR per SD (145.5 g/day) increase, 1.28; 95 % CI, 1.06, 1.55).

No statistically significant association was found between low-fat dairy and CVD mortality (Table 2). Furthermore, no statistically significant association was found between fermented dairy and CVD mortality and between cheese and CVD mortality.

Adjusting for energy according to the residual method of Walter Willett [25] gave identical results (data not shown).

### Dairy intake and total mortality

There was no significant relationship between any of the dairy categories and total mortality. A significant inverse association was found between low-fat dairy and total mortality, fermented dairy and total mortality, and between cheese and total mortality in the crude model only. After further adjustments, the HRs did not remain statistically significant (Table 3).

**Table 3:** Hazard ratios (HRs) and 95 % CIs of total mortality per SD increase of dairy intake

Total mortality (*n* = 403)*				
	Model 1^a^	Model 2^b^	Model 3^c^	Model 4^d^
Total dairy^e^	0.91 (0.82, 1.01) *P* = 0.06	**0.89 (0.81, 0.99)** *P* = **0.03**	0.93 (0.83, 1.04) *P* = 0.23	0.97 (0.86, 1.09) *P* = 0.61
High-fat dairy^f^	1.01 (0.91, 1.11) *P* = 0.93	0.98 (0.89, 1.08) *P* = 0.71	1.00 (0.90, 1.11) *P* = 0.98	1.02 (0.90, 1.16) *P* = 0.72
Low-fat dairy^g^	**0.90 (0.81, 1.00)** *P* = **0.04**	0.90 (0.82, 1.00) *P* = 0.05	0.94 (0.85, 1.05) *P* = 0.27	0.96 (0.86, 1.08) *P* = 0.50
Milk and milk products^h^	0.92 (0.83, 1.02) *P* = 0.11	**0.90 (0.81, 0.99)** *P* = **0.04**	0.94 (0.84, 1.05) *P* = 0.25	0.97 (0.86, 1.09) *P* = 0.61
Milk^i^	0.96 (0.87, 1.06) *P* = 0.39	0.92 (0.83, 1.01) *P* = 0.09	0.96 (0.86, 1.07) *P* = 0.43	0.96 (0.86, 1.08) *P* = 0.53
Fermented dairy^j^	**0.87 (0.78, 0.97)** *P* = **0.01**	0.92 (0.83, 1.02) *P* = 0.12	0.95 (0.85, 1.06) *P* = 0.33	0.98 (0.87, 1.11) *P* = 0.77
Cheese^k^	**0.84 (0.75, 0.94)** *P* = **0.002**	0.90 (0.80, 1.00) *P* = 0.06	0.93 (0.83, 1.05) *P* = 0.26	0.96 (0.84, 1.09) *P* = 0.51

The SDs were 271 g/day for total dairy, 179 g/day for high-fat dairy, 260 g/day for low-fat dairy, 268 g/day for milk and milk products, 202 g/day for milk, 182 g/day for fermented dairy, and 22 g/day for cheese

Bold values are statistically significant (*P* < 0.05)

* Due to missings on confounder variables, the number of cases in model 3 is 380 (total, 1,848), and in model 4 this is 332 (total, 1,637)

^a^Unadjusted

^b^Adjusted for age and sex

^c^Adjusted for age, sex, BMI, smoking, educational level, total energy intake, alcohol consumption

^d^Adjusted as for model 1 with additional adjustment for physical activity and intake of meat, fish, bread, vegetables, fruit, coffee, and tea

^e^Includes all dairy products except butter

^f^Defined as all milk products with a fat content >2.0/100 g or cheese products with a fat content >20/100 g

^g^Defined as all milk products with a fat content <2.0/100 g or cheese products with a fat content <20/100 g

^h^Includes all kinds of milk, yoghurt, coffee creamer, curd, cream, porridge, and pudding (both low-fat and high-fat)

^i^Includes all milk: skimmed, semi-skimmed, and whole milk

^j^Includes all fermented products, such as yogurt, buttermilk, curds, and cheese products

^k^Includes soft cheese and hard cheese (both low-fat and high-fat)

## Discussion

In this cohort study among 1956 Dutch men and women aged 50–75 years, a positive association was found between high-fat dairy intake and the risk of CVD mortality.

Several observational studies have evaluated the association between dairy product intake and CVD outcomes [4, 8, 9, 11–14, 24, 26, 27]. The results of these studies were inconsistent, possibly because of the heterogeneity of the population and the differences in design and dietary assessment methodology used by the various studies, as well as the exposures and outcomes studied. Dairy intake is a rather heterogeneous exposure, and little is known about specific types of dairy foods in relation to CVD.

Total dairy was not associated with CVD mortality and all-cause mortality in this study. A previous study within the Hoorn Study [24] showed that the consumption of high-fat dairy was significantly inversely associated with cardiovascular risk factors such as BMI and lipid levels, which may seem inconsistent with our current results. However, the cross-sectional associations are likely to be disturbed by reversed causation [24], a phenomenon which is excluded in longitudinal studies. Our current results on mortality are in line with an earlier prospective analysis of the Hoorn Study, where no association was found between total dairy intake and changes in cardiovascular risk factors [27]. No distinction was made between different types of dairy in this study.

In the present study, high-fat dairy intake was associated with an increased risk of CVD mortality. Dairy foods, and especially high-fat dairy, contribute to the intake of saturated fat. Especially longer chain saturated fat has previously been associated with higher incidence of CHD [28]. Moreover, saturated fatty acids raise the LDL-cholesterol concentration in the blood, which is one of the main risk factors for CVD. A recent study of Goldbohm et al. [29] found that dairy fat intake was associated with slightly increased all-cause and ischemic heart disease mortality rates. Further evidence to support our positive results on high-fat dairy and CVD mortality came from ecological studies [30, 31] and controlled feeding studies finding associations on LDL-cholesterol [32, 33]. To explore whether the positive association between high-fat dairy and CVD mortality can be attributed to fat or more sugar containing products, we split our analyses by desserts. Increased risks for CVD were only found in the “non-desserts” category. This strengthened our suggestion that it is potentially the high amount of fat that causes this high risk of CVD mortality. On the other hand, additional adjustment for saturated fat moderately reduced the association between high-fat dairy and CVD mortality, but remained statistically significant. It is therefore very well possible that besides saturated fat, there are other, unknown, components or mechanisms that could exert an effect on the risk of CVD mortality [34]. The consumption of high-fat dairy might for example reflect more unhealthy lifestyle or dietary pattern, which was not all measured. Residual confounding cannot be completely ruled out. Furthermore, when physical activity, the confounder with the largest number of missing values (*n* = 255), was removed from the final model, the hazard ratio became lower (HR, 1.24; 95 % CI, 1.03, 1.49), but remained statistically significant. Caution is therefore needed with the conclusion that each SD increase in high-fat dairy intake would be associated with a 32 % higher risk of CVD mortality.

There is growing evidence for a protective effect of low-fat dairy on blood pressure [13–15]. The mechanism by which especially low-fat dairy exerts a beneficial effect on blood pressure remains to be established. Vitamin K2, naturally present in fermented dairy products, has been recently suggested to decrease aortic calcification and CHD [35, 36].

In this study, fermented dairy and low-fat dairy were not statistically significantly associated with CVD mortality or all-cause mortality. Bernstein et al. [37] reported inverse associations between low-fat dairy and CHD only after 26 years of follow-up. It might be possible that the beneficial effect of fermented and low-fat dairy on CVD becomes visible after longer exposure and thus longer follow-up time.

Several strengths and limitations of our study need to be addressed. In the Netherlands, the intake of dairy products is naturally high, with a wide variety of dairy products that are consumed. To put this in perspective, in the most recent European Nutrition and Health report, the highest consumption of milk- and dairy-based products was reported in countries like Norway (522 g/day), Finland (437 g/day), the Netherlands (388 g/day), and United Kingdom (320 g/day), whereas intake was low in Austria (171 g/day), Poland (181 g/day), and the Czech Republic (186 g/day) [38]. In our data, the mean intake of dairy (466 g/day) was higher than in this report (mean dairy intake 266 g/capita/day over 16 European countries) with a large variation, which enabled us to examine the relationship between dairy food consumption and CVD over a broad range of intake.

A possible limitation is the estimation of dairy intake used in the analysis, which was derived from a semi-quantitative FFQ. The self-report of usual dietary intake could lead to misclassification. However, the FFQ was validated by comparison with a modified dietary history. Pearson correlation coefficients for estimates from the questionnaire and dietary history were on average 0.71 (range, 0.65–0.78) for macronutrients and 0.66 (range, 0.36–0.81) for vitamins and minerals [23]. Although the FFQ was not validated for dairy intake, high correlations were found for protein and calcium (0.69 and 0.75, respectively). Moreover, in the Netherlands, dairy is consumed on typical moments during the day, often during breakfast and/or lunch and after a hot meal, making it easier for the participants to recall the usual dairy intake.

A limitation of our study is that only dairy consumption at baseline was investigated, and not during follow-up. Participants may have changed their dietary pattern during the study. Although it cannot be assumed, 50- to 75-year-old adults may be less likely to change nutritional habits.

Another limitation is that we had a limited number of CVD and all-cause mortality cases. Therefore, we could not carry out analyses for dairy consumption by tertiles and presented continuous analyses per SD. Furthermore, no information on CVD morbidity was available. The participants had to give permission to access their hospital records, which only a small number of people did. It was therefore only possible to assess the relationship with mortality, and not with morbidity.

At last, a remark should be made about the age of the population. This study population was somewhat older than other cohorts, with an age between 50 and 75 years at baseline. Caution is needed with generalizing these results to a younger population.

In this Dutch prospective cohort study, the intake of high-fat dairy products was associated with an increased risk of CVD mortality.

Based on the results of this study, it can indeed be recommended to rather consume low-fat dairy instead of high-fat dairy in order to reduce the risk of CVD. However, to draw valuable conclusions, more research is needed in larger prospective studies with sufficient cases of stroke and CHD.
